# Carboxyethylsilanetriol-Functionalized Al-MIL-53-Supported Palladium Catalyst for Enhancing Suzuki–Miyaura Cross-Coupling Reaction

**DOI:** 10.3390/molecules30030656

**Published:** 2025-02-01

**Authors:** Yucang Liang, Xin Ning, Yanzhong Zhen

**Affiliations:** Institut für Anorganische Chemie, Eberhard Karls Universität Tübingen, Auf der Morgenstelle 18, 72076 Tübingen, Germany

**Keywords:** microporous Al-metal–organic framework, structure functionalization, palladium heterogeneous catalysis, C-C cross-coupling reaction, reusability

## Abstract

The application of metal–organic frameworks (MOFs) has attracted increasing attention in organic synthesis. The modification of MOFs can efficiently tailor the structure and improve the property for meeting ongoing demand in various applications, such as the alteration of gas adsorption and separation, catalytic activity, stability, and sustainability or reusability. In this study, carboxyethylsilanetriol (CEST) disodium salt was used as a dual-functional ligand for modified Al-MIL-53 to fabricate CEST-functionalized Al-MIL-53 samples through a hydrothermal reaction of aluminum nitrate, terephthalic acid, and CEST disodium salt by varying the molar ratio of CEST to terephthalic acid and keeping a constant molar ratio of Al^3+^/-COOH of 1:1. The structure, composition, morphology, pore feature, and stability were characterized by XRD, different spectroscopies, electron microscopy, N_2_ physisorption, and thermogravimetric analysis. With increasing CEST content, CEST-Al-MIL-53 still preserves an Al-MIL-53-like structure, but the microstructure changed compared with pure Al-MIL-53 due to the integration of CEST. Such a CEST-Al-MIL-53 was used as the support to load Pd particles and afford a catalyst Pd/CEST-Al-MIL-53 for Suzuki–Miyaura C-C cross-coupling reaction of aryl halides and phenylboronic acid under basic conditions. The resulting Pd/CEST-Al-MIL-53 showed a high catalytic activity compared with Pd/Al-MIL-53, due to the nanofibrous structure of silicon species-integrated CEST-Al-MIL-53. The nanofiber microstructure undergoes a remarkable transformation into intricate 3D cross-networks during catalytic reaction, which enables the leachable Pd particles to orientally redeposit and inlay into these networks as the monodisperse spheres and thereby effectively preventing Pd particles from aggregation and leaching, therefore demonstrating a high catalytic performance, long-term stability, and enhanced reusability. Obviously, the integration of CEST into MOFs can effectively prevent the leaching of active Pd species and ensure the re-deposition during catalysis. Moreover, catalytic performance strongly depended on catalyst dosage, temperature, time, solvent, and the type of the substituted group on benzene ring. This work further extends the catalytic application of hybrid metal–organic frameworks.

## 1. Introduction

Metal–organic frameworks (MOFs) have attracted increasing attention due to the diversity of structures and morphologies [1,2] fabricated by various multifunctional linkers, [3,4,5,6], tunable microporous to hierarchical pore structures, ultrahigh porosity and high specific surface areas, chemical/thermal and mechanical stabilities [7], and wide applications in the fields of catalysis [8], gas separations and purification [9,10,11], the discrimination of pollutants [12], and energy storage and conversion [13,14,15]. Such MOFs can be constructed by hydro/solovothermal technique, self-assembly approach, even external-stimuli-induced structure transformation [16]. In these MOFs, aluminum-based metal–organic frameworks have been explored extensively, thanks to low toxicity, low cost, abundant reserves in the earth, high sustainability, and wide applications [17,18]. First, a crystalline porous aluminum terephthalate compound MIL-53 was prepared by the hydrothermal reaction of aluminum nitrate nonahydrate (Al(NO_3_)_3_·9H_2_O and 1,4-benzenedicarboxylic acid (H_2_BDC) (MIL = Matériaux Institut Lavoisier) [19,20]. A facile and simple method was developed to prepare Al-MIL-53 at room temperature [21]. The thermal chemistry behavior and crystallization mechanism of Al-MIL-53 were explored [22,23,24].

Due to the convenient preparation and functionalization of Al-MIL-53, it is widely applied in the fields of adsorption, separation and purification, [9,10,11,12,19,25,26] and catalytic reactions. Particularly, Al-MIL-53 supports the preparation of a new class of heterogeneous catalysts for catalytic applications. These catalytic reactions include aldehyde self-condensation [27], aldol condensation of acetaldehyde [28], aldol reaction of 4-nitrobenzaldehyde and acetone [29], CO_2_ reduction [30], selective sulfoxidation reaction [31], methanol dehydration [32], and photocatalytic H_2_O_2_ generation [33,34]. Note that metallic Pd in Pd-loaded Al-MIL-53 plays crucial role for the C-C cross-coupling reaction to enhance the catalytic activity and confirm the effectiveness of the catalytic performance [35,36]. Cao and co-workers loaded Pd nanoparticles onto MIL-53(Al) and MIL-53(Al)-NH_2_ to afford catalysts Pd/MIL-53(Al) and Pd/MIL-53(Al)-NH_2_, and only the latter was used to evaluate its catalytic performance for Suzuki–Miyaura coupling reactions of aryl halides and phenylboronic acid [37]. Kleist and co-workers performed Heck-type C-C coupling reactions of bromo- or chlorobenzene with styrene over Pd/MIL-53(Al)-NH_2_ [38]. In addition, Pd/MIL-53(Al) can also be used as a catalyst for iodobenzene methoxycarbonylation [39]. More recently, Pd/MIL-53(Al) was used as a catalyst for direct H_2_O_2_ synthesis, unraveling structure–property relationships [40]. These recent investigations have further solidified the remarkable catalytic efficiency and recyclability of Pd/Al-MIL-53, demonstrating the success of optimizing both synthetic and catalytic conditions to achieve a higher level of sustainability. Such advancements highlight significant strides in overcoming previous limitations, positioning Pd/Al-MIL-53 as a promising candidate for next-generation catalysts, particularly for industrial applications. However, despite these optimizations and strategies addressing several challenges, the persistent issue of Pd leaching remains a significant hurdle in heterogeneous catalysis. The loss of Pd during catalytic cycles can not only diminish the catalyst’s performance but also reduce its economic viability and environmental sustainability. Hence, the modification of Al-MIL-53 as a support material is an effective and critical approach to mitigate or prevent undesirable loss of Pd during catalytic cycles, offering a pathway to further enhance catalyst efficiency and stability. By tailoring the structural properties of Al-MIL-53 (such as its surface area, pore structure, and metal coordination) to strengthen the interaction between palladium and the framework, Pd loss can be effectively minimized during catalytic processes. Such modification strategy is becoming increasingly vital in ensuring longevity and sustainability of the catalyst, which are essential for its practical application in large-scale industrial applications. As such, the continued refinement of Al-MIL-53-based catalysts holds great promise for achieving a more efficient, cost-effective, and environmentally friendly catalytic process, advancing the development of high-performance catalysts for future chemical manufacturing and industrial applications.

Based on above-mentioned modification strategy, in this work, three carboxyethylsilanetriol (CEST)-functionalized Al-MIL-53 samples are successfully prepared by adjusting the molar ratio of CEST to terephthalic acid under an identical molar ratio of Al^3+^/-COOH of 1:1 through a hydrothermal reaction. With the increasing CEST content, C contents in CEST-Al-MIL-53 (**1**–**3**) remarkably decreased, but all three samples still preserved intact Al-MIL-53-like crystalline structures confirmed by XRD patterns. IR and NMR spectroscopies corroborated the integration of CEST into the Al-MIL-53 framework. N_2_ physisorption determines their microporous structure, and the integrated CEST group in Al-MIL-53 makes the specific surface area greatly decline while also altering the morphology from an irregular blocky shape to a regular nanofiber. Such a thermally stable CEST-Al-MIL-53 was used as a support to load the Pd nanoparticle for fabricating the Pd/CEST-Al-MIL-53 catalyst. The resulting Pd/CEST-Al-MIL-53 was applied in the Suzuki–Miyaura cross-coupling reaction of benzene halide and phenylboronic acid and showed an excellent catalytic performance and high reusability compared with Pd/Al-MIL-53, revealing the crucial role of the CEST group for stabilizing Pd nanoparticles.

## 2. Results and Discussion

### 2.1. Synthesis and Characterization of CEST-Al-MIL-53 with Different CEST Contents

**Figure 1:**
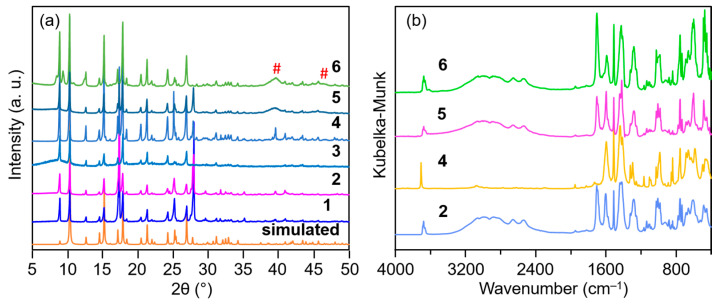
(**a**) XRD patterns of samples CEST-Al-MIL-53 (**1**–**3**) with various CEST contents, Al-MIL-53 (**4**), Pd/CEST-Al-MIL-53 (**5**), Pd/Al-MIL-53 (**6**), and simulated as-synthesized Al-MIL-53. The diffraction peaks of crystalline metallic Pd are marked as “#”. (**b**) Infrared resonance spectra of sample CEST-Al-MIL-53 (**2**), Al-MIL-53 (**4**), Pd/CEST-Al-MIL-53 (**5**), and Pd/Al-MIL-53 (**6**).

**Figure 2:**
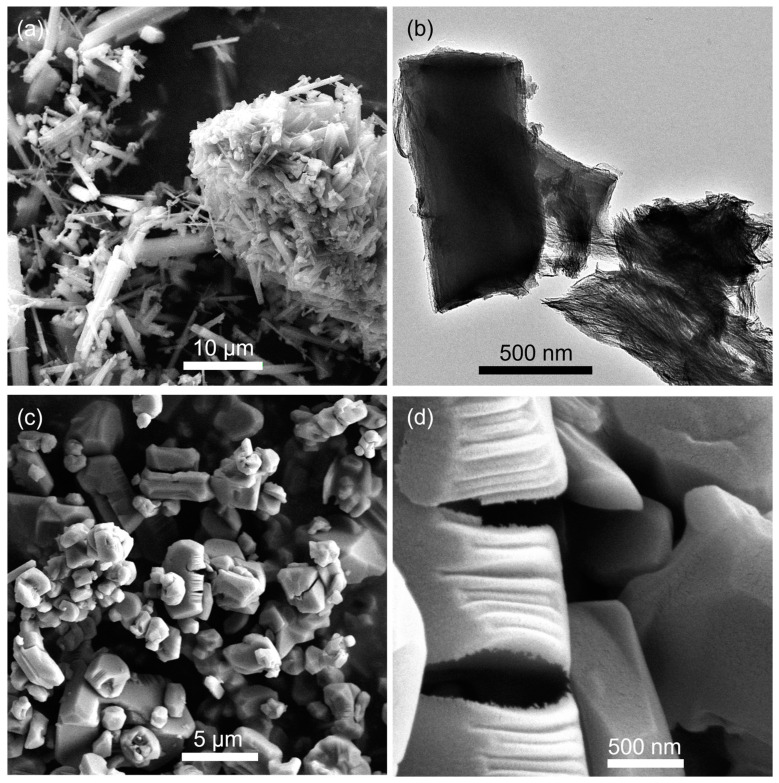
(**a**) SEM and (**b**) TEM image of sample **2**. (**c**,**d**) SEM images of sample **4** with different magnifications.

The integration of a silica subunit into a metal–organic framework probably promotes the thermal stability of coordination frameworks. Herein, carboxyethylsilanetriol (CEST) disodium salt was used to replace partial terephthalic acid in the preparation of Al-MIL-53 [20] and fabricate CEST-functionalized Al-MIL-53 containing ≡Si-(CH_2_)_3_COO-Al subunit. CEST disodium salt first reacted with Al^3+^ for 10 min, and then terephthalic acid was added. After a hydrothermal treatment at 160 °C for 24 h, silicon-species-hybridized samples CEST-Al-MIL-53 (**1**–**3**) were obtained. As can be seen in Figure 1a, the XRD patterns of samples **1**–**3** clearly show the characteristic diffraction peaks as the simulated as-made Al-MIL-53, corroborating the formation of the Al-MIL-53-like structure. With the increasing molar ratio of CEST to terephthalic acid, samples **1**–**3** do not show any significant change in the position and intensity of diffraction peaks. As a representative, CEST-Al-MIL-53 (**2**) was chosen to measure the SEM and TEM images. The results exhibit that sample **2** had a rod-like morphology with various widths and lengths (Figure 2a). The TEM image verified that such rods were composed of nanofibers and cross-connected together to form 3D networks (Figure 2b). Note that Al-MIL-53 (**4**) prepared according to the literature [20] showed an irregular blocky morphology (Figure 2c,d), implying that the integration of CEST alters the morphology of the final microporous materials. In addition, based on an elemental analysis and XRD patterns, the molecular structures of CEST-Al-MIL-53 (**1**) and (**2**) are suggested as (HO)Al(O_2_CC_6_H_4_CO_2_)_0.95_(O_2_CCH_2_CH_2_Si(OH)_3_)_0.1_·(HO_2_CC_6_H_4_CO_2_H)_0.7_ and (HO)Al(O_2_C-C_6_H_4_CO_2_)_0.9_(O_2_CCH_2_CH_2_Si(OH)_3_)_0.2_·(HO_2_CC_6_H_4_CO_2_H)_0.7_, respectively. For CEST-Al-MIL-53 (**3**), it is difficult to propose a rational molecular structure due to high CEST content and Si-O-Si subunit; as a result, carbon contents in **3** remarkably decreased compared with **1** and **2**, but the Al-MIL-53-like crystalline structure still exists in **3** (Figure 1a).

In order to further corroborate the integration of CEST ligand, infrared resonance (IR) spectroscopy of CEST-Al-MIL-53 (**2**) was measured. As shown in Figure 1b, the characteristic bands at 1702 cm^−1^, 2881~2987 cm^−1^, 2536, and 2659 cm^−1^ are observed in the IR spectra, which are assigned to C=O and C-H bonds from the CEST group, while the stretching vibrations of C-H and C-C bonds from the benzene ring appeared at 3060 cm^−1^ and 1480~1595 cm^−1^, respectively. Note that O-H from the -AlOH and -SiOH group is also found at 3704 cm^−1^. In addition, the bending vibration of the C-H bond attributable to free terephthalic acid or coordinated terephthalate group appeared at 1287 cm^−1^. All these vibrations directly corroborate the existence of the terephthalate, -SiCH_2_CH_2_CO_2_^−^ group, and uncoordinated terephthalic acid in CEST-Al-MIL-53. Moreover, the weak Si-O-Si stretching vibration was also observed at 1105 and 1130 cm^−1^, further confirming the condensation of terminal silanol groups.

**Figure 3:**
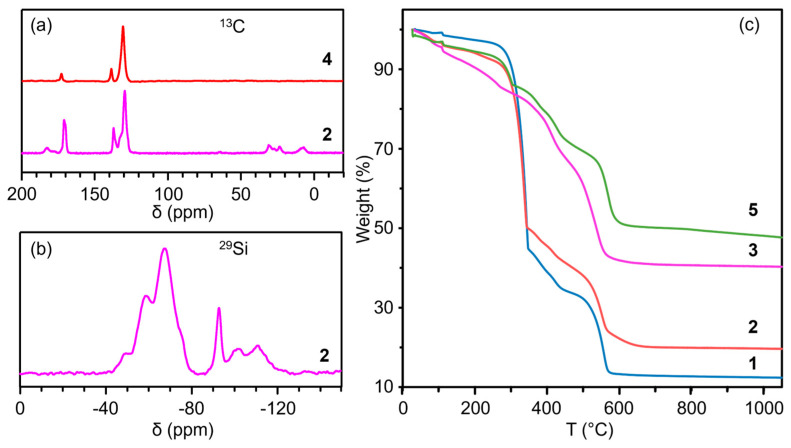
(**a**) ^13^C NMR spectra of CEST-Al-MIL-53 (**2**) and Al-MIL-53 (**4**); (**b**) ^29^Si NMR spectrum of CEST-Al-MIL-53 (**2**); (**c**) TGA curves of samples CEST-Al-MIL-53 (**1**–**3**) and Pd/CEST-Al-MIL-53 (**5**).

To further verify the existence of the CEST group and its intact structure in Al-MIL-53, solid state ^13^C and ^29^Si NMR spectra for CEST-Al-MIL-53 (**2**) were measured. As shown in Figure 3a, ^13^C NMR spectrum clearly showed not only three characteristic signals at 131.5, 139.2, and 173.0 ppm belonging to carbon atoms from terephthalate but also typical carbon atom signals at 9.0, 29.1, 32.9, 181.3, and 184.8 ppm from functional groups ≡SiCH_2_CH_2_CO_2_^−^ and CH_3_CH_2_CO_2_^−^ derived from the cleavage of partial C-Si bonds. The uncoordinated terephthalic acid was not detected by the ^13^C NMR spectrum. Note that the solid-state ^29^Si NMR spectrum of sample **2** not only indicated three characteristic peaks at −48.7, −58.6, and −67.5 ppm, which can be attributed to organosilicon species, T^1^ [(HO)_3_SiCH_2_CH_2_CO_2_^−^)], T^2^ [(SiO)_2_(HO)SiCH_2_CH_2_CO_2_^−^], and T^3^ [(SiO)_3_SiCH_2_CH_2_CO_2_^−^], respectively, but also showed three weak silicon species Q^n^ ([Si(OSi)_n_(OH)_4-n_], *n* = 2, 3, 4) appeared at −92.7, −101.8, and −110.9 ppm (Figure 3b), directly confirming the integration of functional group ≡SiCH_2_CH_2_CO_2_^−^ into the Al-MIL-53 frameworks, the cleavage of partial C-Si bond, and further condensation of the silanol group during the hydrothermal reaction. Based on these analyses, strictly speaking, sample **2** is a typical inorganic–organic hybrid material, SiO_2_-integrated CEST-Al-MIL-53. Probably, the integration of the silicon species into CEST-Al-MIL-53 makes MOF become more stable and useful in catalysis. Note that the ^13^C NMR spectrum of Al-MIL-53 (**4**) only exhibits three characteristic carbon signals from the coordinated terephthalate group (Figure 3a). The results from the ^13^C and ^29^Si NMR spectra are in good agreement with the characteristic functional group characterized by the IR spectra.

To verify the thermal stability of CEST-Al-MIL-53 with various contents of the CEST group, a thermogravimetric analysis (TGA) was performed. As shown in Figure 3c, the TGA curves show that the thermal stability of CEST-Al-MIL-53 strongly depends on the CEST contents. For CEST-Al-MIL-53 (**1**) with low CEST content, the weight was reduced by about 4.0% when the temperature was less than 265 °C, which corresponds to the loss of adsorbed water molecules. With increasing temperatures in the range of 265–350 °C, the weight was decreased by about 51.4%. This belongs to the decomposition of the functional group –CH_2_CH_2_COO^−^ and free terephthalic acid. With further increasing temperatures in the range of 350–570 °C, the CEST-modified Al-MIL-53 frameworks completely collapsed, and the weight was reduced by about 30.6%. Finally, the residue about 14% should be an oxide of aluminum and silicon. With increasing CEST contents, the CEST-Al-MIL-53 (**2**) experiences a similar process, but weight loss is different in every stage, about 8.4% below 265 °C, about 41.8% in the temperature range of 265–350 °C, about 25.8% in the temperature range of 350–570 °C, about 3.4% in the range of 570–645 °C, and about a constant 20.6% residue (alumina and silica) above 645 °C. With further increasing CEST contents, CEST-Al-MIL-53 (**3**) slowly decomposed, and lost about 14.0 wt% weight below 265 °C, about 42.2% in the temperature range of 265–560 °C, and preserved about 43.5% constant residue with alumina and silica over 560 °C. Obviously, the increased CEST leads to the increase in final residue due to the generation of more silicas. These results demonstrate that CEST-Al-MIL-53 is stable below 265 °C except from CEST-Al-MIL-53 (**3**). The results from TGA are in good agreement with the molecular structure of CEST-Al-MIL-53 (**1**, **2**) suggested.

**Figure 4:**
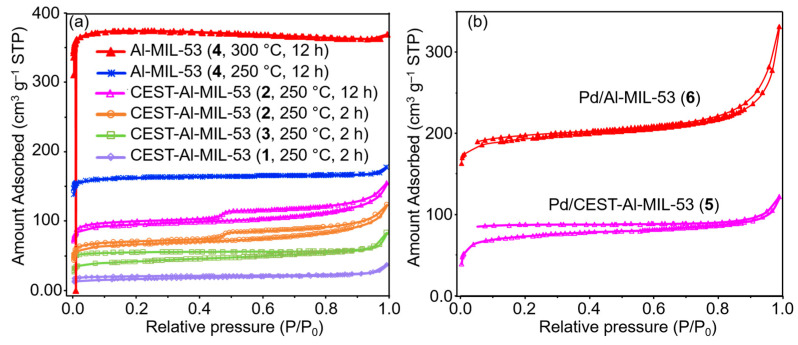
(**a**) N_2_ physisorption isotherms of CEST-Al-MIL-53 (**1**–**3**) and Al-MIL-53 (**4**) activated at different temperatures. (**b**) N_2_ physisorption isotherms of Pd/CEST-Al-MIL-53 (**5**) and Pd/Al-MIL-53 (**6**).

To well demonstrate the micropore structure of CEST-Al-MIL-53 (**1**–**3**) and pure Al-MIL-53 (**4**), N_2_ adsorption–desorption isotherms were measured. Based on TGA data, prior to the N_2_ physisorption analysis, the sample was degassed at different temperatures (250 °C and 300 °C). As can be seen in Figure 4a, samples **1**–**4** indicate type I isotherm curves, showing characteristic microporous materials. Note that with the integration of the functional group CEST and gradually increasing contents in the Al-MIL-53 framework, the micropore structure was filled with a bridged Si-O-Si subunit or terminal ≡Si-OH groups, which causes a remarkable decline in the specific surface area compared to Al-MIL-53 (**4**). Surprisingly, when CEST-Al-MIL-53 (**2**) was degassed at 250 °C and 300 °C, respectively, N_2_ physisorption shows characteristic hierarchical pore materials due to type I and IV isotherms. With increasing activated temperature, the contribution of micropore-to-pore volume is dominant. Pore parameters of all samples are listed in Table 1.

### 2.2. Synthesis and Characterization of Pd/CEST-Al-MIL-53 (***5***) and Pd/Al-MIL-53 (***6***)

**Figure 5:**
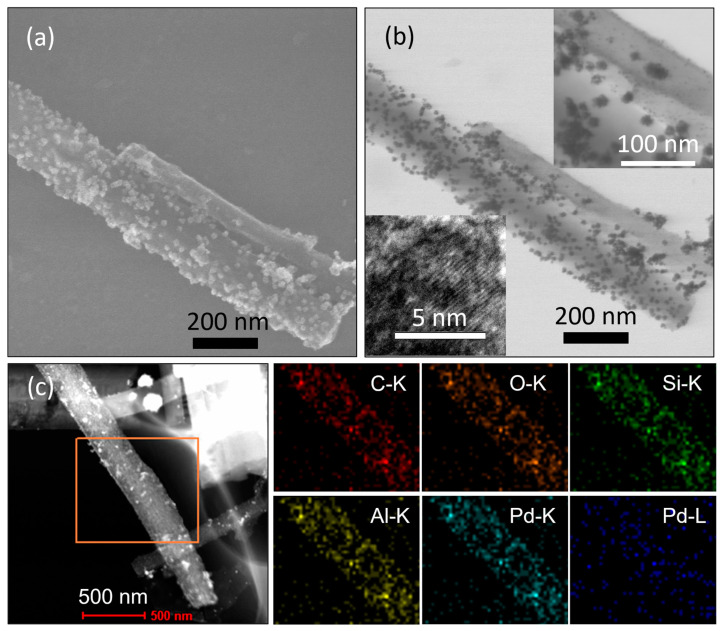
(**a**) SEM and (**b**) TEM images of sample Pd/CEST-Al-MIL-53 (**5**). The inset is HRTEM images in (**b**). (**c**) Scanning electron microscopy and EDX spectroscopic elemental mappings of C, O, Si, Al, and Pd in sample **5**.

On the basis of the pore parameter and structure of the CEST-functionalized Al-MIL-53 (**1**–**3**), CEST-Al-MIL-53 (**2**), with a mild molar ratio of CEST to terephthalic acid, was chosen as a support for loading Pd nanoparticles. An expected amount of K_2_PdCl_4_ dissolved in water was added into an aqueous suspension of CEST-Al-MIL-53 (**2**), and this suspension was stirred at an ambient temperature for 20 min to let Pd^2+^ homogeneously diffuse into the micropore channel of MOF frameworks. Afterwards, NaBH_4_ as a reductant was added to perform the reduction of Pd^2+^ to Pd^0^ and in situ deposit into/onto CEST-AL-MIL-53 frameworks. The resulting black solid Pd/CEST-Al-MIL-53 (**5**) was obtained. As shown in Figure 1a, the XRD pattern of sample **5** not only intactly preserves the characteristic diffraction peaks of CEST-Al-MIL-53 but also clearly exhibites the characteristic diffraction peaks (marked as #) of the crystalline Pd phase with face-centered cubic *Fm-3m* symmetry, pointing to the formation of Pd particles. The SEM images shown in Figure 5a indicate that Pd nanoparticles also randomly deposited on the surface of microporous nanofiber-shaped CEST-Al-MIL-53 with the loose cluster-like aggregations (11.8 ± 0.5 nm) and single small particles (2.4 ± 0.2 nm) (Figure 5a,b). Note that such aggregations consisted of small particles of 1–5 nm, and the lattice fringes of crystalline Pd particles can be easily observed (Figure 5b, inset). These kinds of loose cluster-like aggregations and single-particle deposition are probably beneficial for the improvement of the catalytic activity. For such a material, the distributions of all elements were investigated using elemental mapping (Figure 5c), directly exhibiting a relatively uniform integration of the CEST ligand into the Al-MIL-53 frameworks during synthesis process and a high dispersion of Pd nanoparticles.

**Figure 6:**
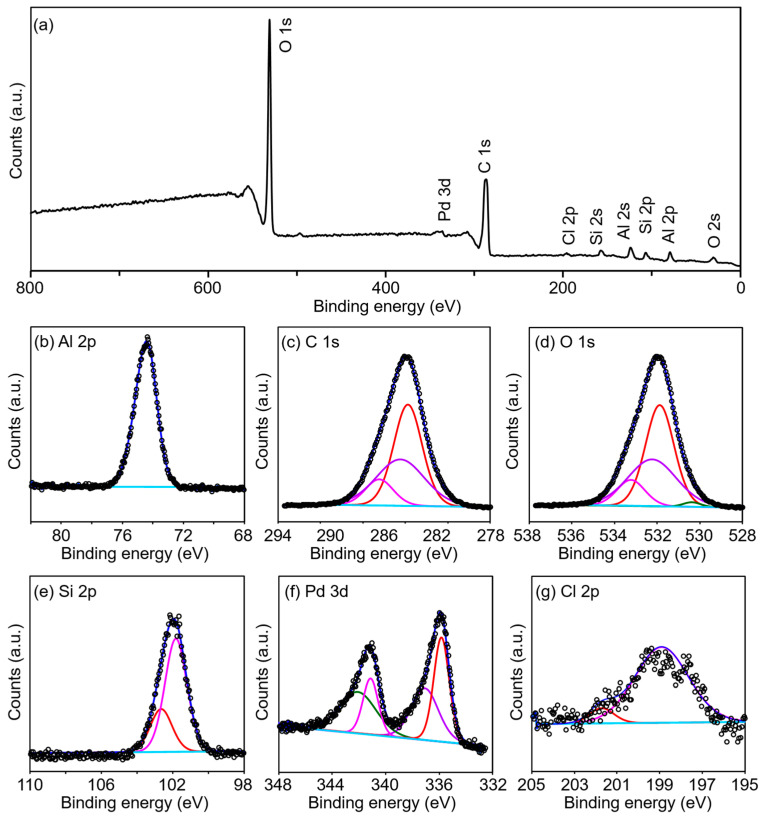
(**a**) XPS survey scan spectrum and high-resolution XPS spectra of sample Pd/CEST-Al-MIL-53 (**5**) for (**b**) Al 2p, (**c**) C 1s, (**d**) O 1s, (**e**) Si 2p, (**f**) Pd 3d, and (**g**) Cl 2p.

XPS spectroscopy is often used to detect chemical composition and the valence state of every element. Here, a representative microporous material Pd/CEST-Al-MIL-53 (**5**) was chosen to measure the XPS spectrum. The XPS survey scan spectrum is shown in Figure 6a. The characteristic peaks of all elements Al, C, O, Si, and Pd are clearly observed. This result is in good agreement with the actual composition in the CEST-Al-MIL-53. High-resolution XPS spectra for Al 2p, C 1s, O 1s, Si 2p, and Pd 3d are shown in Figure 6b–f. The binding energy of Al 2p is located at 74.4 eV. C 1s XPS can be fitted into three peaks, which are attributed to the binding energies of C 1s of C-Si (284.6 eV) from silane, of C=C or C-C (286.1 eV), and C=O (288.6 eV) from CEST and terephthalate or free terephthalic acid. For O 1s XPS, the fitted four peaks located at 530.4, 531.9, 532.2, and 533.2 eV, respectively, are in good accordance with the binding energies of O 1s from O-C=O, -C-Si-O-, -O-Si-O-, and Al/Si-OH fragments. Si 2p XPS from the CEST group in Pd/CEST-Al-MIL-53 (**5**) indicated two peaks at 102.7 and 101.8 eV; the former is the binding energy of Si from the terminal-condensed O-Si-O structure, and the latter belongs to Si in C-Si from ≡SiCH_2_CH_2_CO_2_^−^ group. The appearance of the Si XPS directly verifies the successful integration of the CEST group into the Al-MIL-53 frameworks. Note that the peak area of Si 2p from the O-Si-O frameworks is lower than that of silane due to that the O-Si-O fragment derived from the cleavage of partial Si-C bonds of the silane group and further condensation of silanol.

The Pd 3d XPS spectrum of the Pd/CEST-Al-MIL-53 (**5**) can be effectively deconvoluted into two distinct sets of peaks. The peaks at 337.1 eV and 342.2 eV correspond to the binding energies of Pd 3d_5/2_ and Pd 3d_3/2_, respectively, characteristic of Pd^2+^ species. On the other hand, the peaks at 335.8 eV and 341.2 eV are in excellent agreement with the binding energies of Pd 3d_5/2_ and Pd 3d_3/2_ for metallic Pd^0^. The presence of Pd^2+^ ions in the Pd/CEST-Al-MIL-53 (**5**) composite is likely attributed to either the incomplete reduction of Pd^2+^ or the oxidation of Pd^0^ nanoparticles when exposed to air. A closer examination of the peak intensities reveals a predominance of metallic Pd species in the material **5**. Furthermore, the high-resolution Cl 2p XPS spectrum clearly shows two prominent peaks at 199.4 eV and 201.7 eV, corresponding to the binding energies of Cl 2p_3/2_ and Cl 2p_1/2_, respectively (Figure 6g). These peaks provide direct evidence that the Pd^2+^ species in Pd/CEST-Al-MIL-53 (**5**) originated from the initially adsorbed and unreduced PdCl_2_ precursor used during the synthesis, confirming the nature of the Pd^2+^ species present in the material. The Pd species in Pd/CEST-Al-MIL-53 (**5**) serve as the active centers for the Suzuki–Miyaura C–C cross-coupling reaction, with the oxidation states of Pd, specifically Pd^0^ and Pd^2+^, playing a crucial role in determining the nature of the catalytic mechanism. The interaction between metallic Pd^0^ or Pd^2+^ species and substrate governs whether the reaction proceeds via a heterogeneous pathway, occurring at the solid–solution interface, or a homogeneous pathway, occurring within the solution phase. This duality in catalytic behavior offers a unique and versatile mechanism that enhances the efficiency and selectivity of the Suzuki–Miyaura reaction and the reusability of the catalyst.

After Pd loading, the pore structure of the Pd/CEST-Al-MIL-53 was also characterized via the N_2_ physisorption. As shown in Figure 4b, it is obvious to find that Pd/CEST-Al-MIL-53 (**5**) still preserves the micropore structure with the type I isotherm. The specific BET and Langmuir surface area are 241 and 340 m^2^ g^−1^, respectively, and the corresponding micropore volume is 0.077 cm^3^ g^−1^. In addition, for Pd/CEST-Al-MIL-53 (**5**), the TGA shows a similar thermal stability as its parent CEST-Al-MIL-53 (**2**) (Figure 3c). As a comparison, the Pd/Al-MIL-53 (**6**) was also prepared. The SEM and TEM images are shown in Figure S1 in Supporting Information. The N_2_ adsorption–desorption isotherm curve is shown in Figure 4b, and the corresponding pore parameters are listed in Table 1.

### 2.3. Pd/CEST-Al-MIL-53-Catalyzed Suzuki–Miyaura Cross-Coupling Reaction

The catalytic performance of the Pd/CEST-Al-MIL-53 sample (**5**) was evaluated using the Suzuki–Miyaura C-C cross-coupling reaction of iodobenzene and phenylboronic acid under basic conditions (5 mg of catalyst, 0.0025 mmol Pd based on Pd content from ICP analysis). First, catalyst dosage effect, reaction time effect, temperature effect, and solvent effect are investigated.

**Figure 7:**
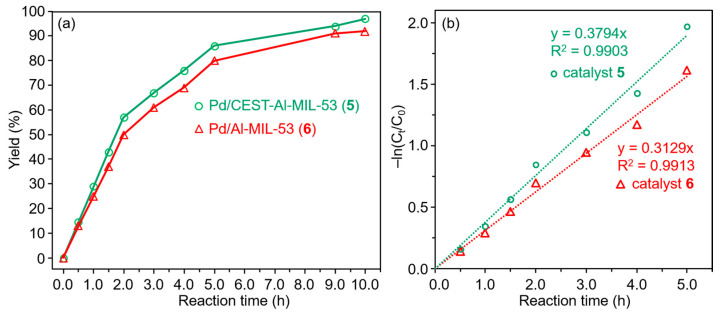
(**a**) Time-dependent catalytic performance of catalysts Pd/CEST-Al-MIL-53 (**5**) and Pd/Al-MIL-53 (**6**) for the C-C cross-coupling reaction of iodobenzene and phenylboronic acid in the presence of K_2_CO_3_ in ethanol; (**b**) the fitted plots of −ln(C_t_/C_0_) versus initial reaction time in the range of 0.5~5 h for catalysts **5** and **6**.

**Figure 8:**
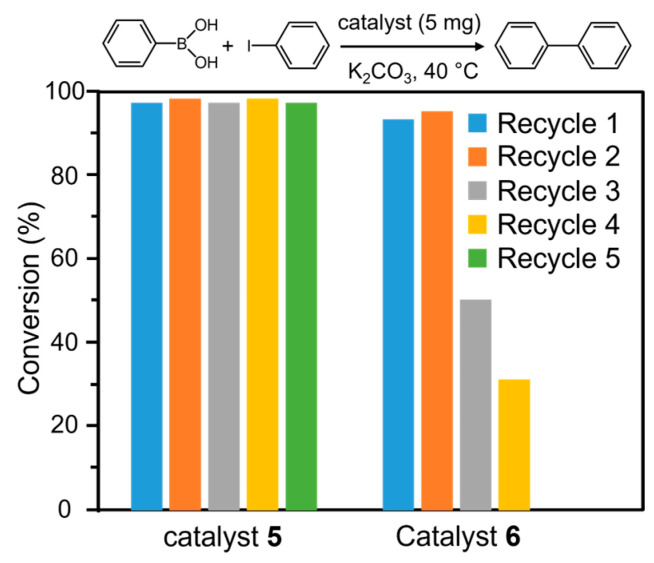
The catalytic conversion of Suzuki–Miyaura C-C cross-coupling reaction of iodobenzene and phenylboronic acid with number of reuse cycles over Pd/CEST-Al-MIL-53 (**5**) and Pd/Al-MIL-53 (**6**).

As listed in Table 2, when 2.5 mg of catalysts **5** was used, conversion only reached 74% within 10 h. When 5 mg and 10 mg of catalyst **5** were, respectively, used, the corresponding conversion is the same (97%) within 10 h. Obviously, the dosage effect of catalyst clearly shows that 5 mg is the best for the Suzuki–Miyaura cross-coupling reaction under other identical conditions. Time-dependent catalytic performance exhibits that the optimal reaction time at 40 °C is 10 h, and conversion reaches 97% (Figure 7a). Moreover, with the increasing reaction temperature from 25 °C to 80 °C, for the same catalytic performance to be reached only requires a short reaction time, pointing to the promotion of reaction kinetics. Based on energy saving, 40 °C is chosen as the reaction temperature. Furthermore, different solvents were used to optimize the reaction solvent; the results indicate that ethanol is an ideal solvent compared with methanol. These investigations are in good agreement with experimental conditions of Pd/Al-MIL-53@HOOC-SBA-15 for C-C cross-coupling reaction [41]. Based on the initial reaction time in the range of 0.5 to 5 h, the plot of −ln(C_t_/C_0_) versus reaction time clearly exhibits a characteristic first-order reaction for catalysts **5** (Figure 7b), and the corresponding initial rate constant is 0.379 h^−1^. As a comparison, the Pd/Al-MIL-53-catalyzed C-C cross-coupling reaction is also a first-order kinetic reaction with a rate constant of 0.313 h^−1^, and the conversion is 92% under the identical condition, implying a low catalytic activity compared with Pd/CEST-Al-MIL-53 (Figure 7). The high catalytic performance of the catalyst Pd/CEST-Al-MIL-53 (**5**) can be attributed to the role of the functional group and silica subunit, the loose cluster-like Pd aggregation composed of small-sized Pd particles with a highly exposed surface and highly dispersed Pd particle deposition in a microporous fiber-like structure. Based on Pd contents calculated using the ICP analysis in the catalyst and initial rate constant, the corresponding turnover frequencies of substrate molecule per millimole Pd in catalysts **5** and **6** are 95.1 h^−1^ and 152.4 h^−1^, respectively. To evaluate the reusability of catalysts **5** and **6**, for the Suzuki–Miyaura C-C cross-coupling reaction, the recyclability was performed (Figure 8). After each catalytic cycle, the catalyst was collected by centrifugation, washed with water several times, and dried in an oven and reused. By comparing catalyst **5** and **6**, obviously, catalyst **5** still preserved a highly catalytic activity about 97% conversion in the fifth run, but the catalytic performance of catalyst **6** markedly declined from 93% in the first run to 31% in the fourth run, confirming the high reusability of catalyst **5**. This result also demonstrates that the integration of CEST and its derived subunit Si-O-Si can efficiently prevent the leaching of Pd-active species. Based on the ICP analysis from catalyst **5** collected after the fifth run and catalyst **6** after the third run, the Pd content is 5.0 wt% for catalyst **5** and 1.1 wt% for catalyst **6**, further verifying the high stability of catalyst 5 and implying serious leaching of most of Pd in catalyst 6 during the catalytic reaction. Obviously, for catalyst **6**, due to leaching of a large amount of active Pd species every recycling run, its catalytic activity quickly declined. After being reused five times for catalyst **5** and three times for catalyst **6**, their morphologies and observable Pd particles were monitored by measuring the SEM and TEM images and further addressed in the following content.

To corroborate the leaching of Pd during catalysis, after Pd/CEST-Al-MIL-53 (**5**)-catalyzed the reaction of iodobenzene and phenylboronic acid was kept at 40 °C for 1 h, all catalysts were separated by centrifugation and filtration to obtain a clear solution, which was kept at 40 °C for 12 h under stirring. The corresponding conversion only increased 2.5% from the GC analysis. Interestingly, we added equivalent amounts of substrates and base into the filtrate and stirred reaction mixture at 40 °C for 12 h. In total, 5 mL of the mixture was taken, centrifugated, and filtered to obtain a clear solution for the GC analysis. The result showed a slightly increased conversion of about 3.0%, indirectly indicating that the leaching of a trace amount of Pd can catalyze the occurrence of the C-C cross-coupling reaction, showing nature of partial homogeneous catalysis (0.29 wt% Pd was leached into solution every recycle from ICP analysis) and revealing the homogeneous–heterogeneous co-catalytic performance or leaching–redeposition mechanism. A slight improvement of conversion is attributed to the addition of base due to removal of insoluble base during centrifugation and filtration. The aforementioned Pd 3d XPS confirmed the existence of Pd^2+^ species in catalyst **5**; the leached Pd content in the solution probably originated from the initial or in situ formed Pd^2+^ during catalytic reaction. Although the leached Pd species can drive C-C coupling reaction, its contribution is limited due to the re-deposition of the leached Pd on support [42,43]; the heterogeneous nature is hence dominant. Similarly, for the Pd/Al-MIL-53 (**6**)-catalyzed C-C cross coupling reaction, Pd leaching almost reached 20 wt% every cycle.

**Figure 9:**
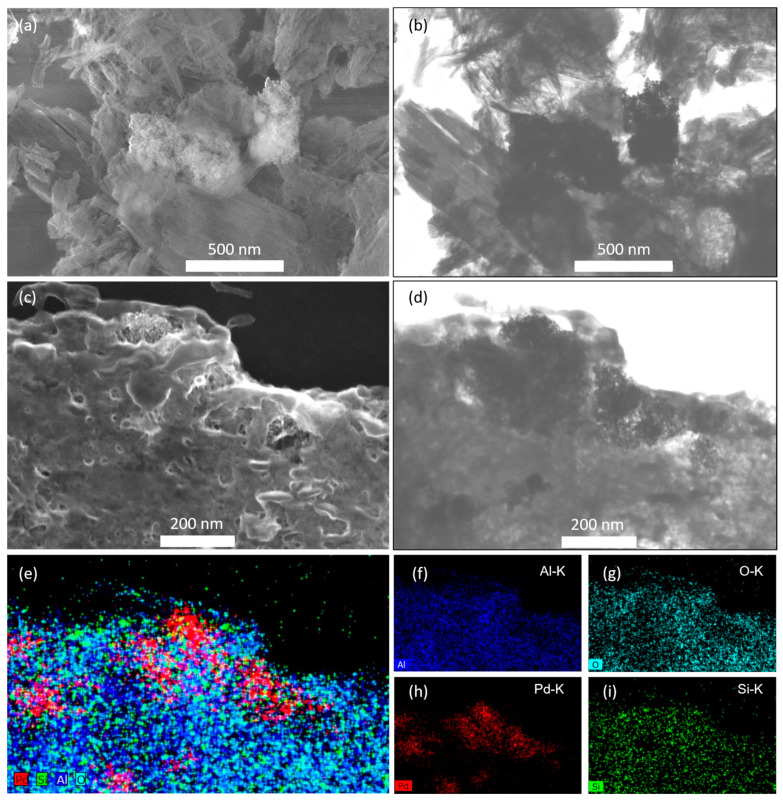
(**a**,**c**) SEM and (**b**,**d**) TEM images of the sample Pd/CEST-Al-MIL-53 (**5**) after the fifth recycled run. (**e**–**i**) The energy-dispersive X-ray spectroscopic elemental mappings of Al, O, Pd, and Si according to image (**c**).

To further unveil the alteration of catalyst on the morphology and composition after catalysis, as shown in Figure 9a–d, the SEM and TEM images of catalyst **5** after the fifth run clearly revealed the existence of Pd particles as two types: Pd aggregations composed of small Pd particles located on external surface of support (Figure 9a,b) and Pd particles as the monodisperse spheres were inlaid into the 3D nanofibrous networks of support (Figure 9c,d). The former derived from the enrichment of original Pd deposition on the external surface, and the latter came from initial Pd loading inside micropore channel of CEST-Al-MIL-53 during catalysis. Note that the inlaid Pd particles can be observed anywhere, which greatly protects from the leaching of Pd particles and makes catalyst **5** to keep highly catalytic performance. In comparison to catalyst **6**, the catalytic activity and reusability of catalyst **5** are much higher, further revealing that the integration of silicon species in CEST-Al-MIL-53 is beneficial to the redeposition of leachable Pd and the formation of monodisperse Pd nanospheres after completion of catalytic reaction. In addition, the condensation of terminal silicon species containing carboxylic acid with a soft, short carbon chain greatly stabilizes the microstructure of CEST-Al-MIL-53 compared to rigid Al-MIL-53.

**Figure 10:**
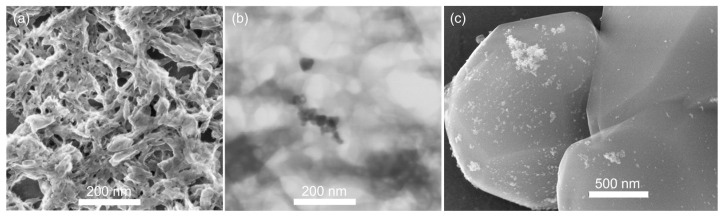
(**a**) SEM and (**b**) TEM image of Pd-Al-MIL-53 after third recycling run. (**c**) SEM image of Pd/Al-MIL-53 before catalysis.

For catalyst **5,** after fifth run, the elemental mappings of Al, O, Pd, and Si were measured and shown in Figure 9e–i, clearly verifying uniform distribution of elements Al, O, and Si and the inlaying of Pd particles into 3D networks composed of nanofibers. Note that the Pd morphology has been changed from previous small particles to spherical particles before and after catalysis. Meanwhile, Pd particles previously located on external surface or into micropore have enriched together to form spherical aggregations or inlaid into microporous nanofiber nets with uniform particle size. As a comparison, the SEM and TEM images of Pd/Al-MIL-53 (**6**) after the third recycling run showed a completely changed morphology (Figure 10a), and a few bulky aggregates of Pd were observed but not much (Figure 10b), which indicates a remarkable difference compared with its parent (Figure 10c and Figure S1).

To further corroborate the formation of a biphenyl product from the reaction of phenyl boronic acid and iodobenzene, for the Pd/CEST-Al-MIL-53 (**5**)-catalyzed C-C cross-coupling reaction for 10 h, an isolated biphenyl product was characterized using ^1^H NMR spectroscopy (^1^H NMR (400 MHz, CDCl_3_, 25 °C). As shown in Figure S2, characteristic chemical shifts of biphenyl are observed at δ = 7.59–7.61 (d, 4H), 7.43–7.47 (t, 4H), and 7.33–7.37 (t, 2H), clearly confirming the formation of product biphenyl.

In addition, as a comparison, the catalytic performance previously reported over Pd-loaded MOFs catalysts in the Suzuki–Miyaura cross-coupling reaction of iodobenzene and phenylboronic acid is listed in Table 3. Obviously, compared with catalysts Pd/MIL-53(Al)-NH_2_ [37], Pd@UIO-66 [44], Pd(II)@MOF-253 [45], Pd/UIO-66-NH_2_ [46], Pd-Sr-MOF-1-PI [47], and Pd-Cu-MOF [48], the catalytic activity for first time has a slight difference, due to the differences in catalyst dosage, reaction temperature, and time. Although the catalysts Pd-Sr-MOF-1-PI and Pd-Cu-MOF seem to be better than Pd/CEST-Al-MIL-53 (**5**), the reaction occurred at a high temperature or with a long reaction time. In fact, catalyst **5** can also reach the same catalytic performance at an identical high temperature and reaction time (Table 2), but in term of reusability or recyclability, the Pd/CEST-Al-MIL-53 (**5**) is the best.

Finally, we extend catalytic application to the C-C cross-coupling reaction of aryl halides with substituted group and phenylboronic acid under the optimized conditions (40 °C, 10 h, ethanol solvent, 5 mg catalyst **5**). As listed in Table 4, bromoaryl derivatives with a strong electron-withdrawing substitute group (such as -NO_2_) indicated a high conversion (entry 1), but 4-bromobenzaldehyde with an electron-withdrawing group showed a low conversion under an identical condition (entry 3). Interestingly, both 4-bromophenol and 4-bromoanisole exhibited a completely different conversion (entries 2 and 5) with an order of -OCH_3_ < -OH, although 4-bromophenol and 4-bromoanisole, respectively, contain an electron-donating hydroxyl and methoxy group. The differences in catalytic performance can be attributed to the following reasons: (1) the stronger electron-donating effect of the −OH group in 4-bromophenol by resonance, which facilitates the Suzuki coupling reaction by stabilizing intermediates and making the C-Br bond more reactive, the stronger the electron-donating nature of the -OH group, which can make the carbon–bromine bond slightly weaker, facilitating the nucleophilic attack by the phenyl anion in the reaction with phenylboronic acid via transmetalation (C_6_H_5_B(OH)_2_ + 4-bromophenol + Pd catalyst → Br-B(OH)_2_ + C_6_H_5_-Pd-C_6_H_4_(OH)). The higher electron-donating effect of -OH group might accelerate the reaction kinetics compared to 4-bromoanisole, leading to a higher yield for 4-bromophenol under the identical reaction conditions; (2) the hydroxyl group in 4-bromophenol may offer less steric hindrance compared to the methoxy group in 4-bromoanisole, further promoting the reaction; (3) the difference in the solubility of 4-bromophenol and 4-bromoanisole in ethanol can also affect the yield: 4-bromophenol can easily dissolve in ethanol compared with 4-bromoanisole due to the potential hydrogen bonding from the hydroxyl group, which might enhance the overall reactivity of 4-bromophenol in the reaction. In addition, the increasing reaction temperature is conducive to the promotion of conversion (entries 4 and 6). On the contrary, bromoaryl derivatives with the electron-donating group by the positively inductive effect showed a very poor conversion (entry 7). Moreover, a chloroaryl derivative (1-chloro-4-nitrobenzene) remarkably passivated the occurrence of this coupling reaction (entry 8). Based on the results of these reactions, the nature of substitute group on the aromatic ring has a markedly different influence on the Suzuki–Miyaura C-C cross coupling reaction with a passivation order of -CH_3_ > -OCH_3_ > -OH and Cl > Br > I.

In addition, for all products, 4-nitrobiphenyl, 4-hydroxybiphenyl, 4-phenylbenzaldehyde, 4-methoxybiphenyl, and 4-methylbiphenyl, shown in Table 4, their ^1^H NMR spectra are shown in Figures S3–S7 (Supporting Information), directly verifying the achievement of catalytic reaction. Note that the ^1^H NMR spectra contain the signals of phenylboronic acid for products 4-hydroxybiphenyl and 4-phenylbenzaldehyde (Figures S4 and S5).

Based on the above-mentioned investigations, although Pd/CEST-Al-MIL-53 has shown a good catalytic activity, long-term recyclability, high selectivity, minimal byproduct formation in the Suzuki–Miyaura C-C cross-coupling reaction, and remarkable potential in the field of environmentally friendly chemistry, a low-cost demand greatly limits its wide application. Hence, a low-cost and highly active Pd-doped transition metal catalyst should be further developed in catalyst design, reaction optimization, and recovery methods to likely further enhance their practical applications, making them a central component of sustainable, green chemical processes.

## 3. Experimental Section

### 3.1. Preparation of Carboxyethylsilanetriol (CEST)-Functionalized Al-MIL-53

CEST-linked Al-MIL-53 (**1**). In a Teflon tube, Al(NO_3_)_3_·9H_2_O (2.701 g, 7.2 mmol) was dissolved in H_2_O (10.0 g), and CEST disodium salt (0.5360 g, 25% in water, 0.68 mmol) was then added. When the mixture was stirred for 10 min, terephthalic acid (0.5413 g, 3.26 mmol) was added, while a molar ratio of Al^3+^/-COOH (from CEST and terephthalic acid) of 1:1 remained. The resulting mixture was continuously stirred for another 10 min, then transferred into an autoclave, heated to 160 °C from ambient temperature in an oven, and kept this temperature for 24 h. Afterwards, the autoclave was naturally cooled down to room temperature, and the solid product was collected via suction filtration, washed with hot water two times, ethanol two times, and then put in a fume hood overnight and dried in an oven at 100 °C for 6 h to afford CEST-Al-MIL-53 (**1**). Elemental analysis (wt%): C, 48.71, H, 3.23, N, 0.24. Based on molar ratio of starting chemicals used and the results of the elemental analysis, the molecular structure is proposed as (HO)Al(O_2_CC_6_H_4_CO_2_)_0.95_(O_2_C-CH_2_CH_2_Si(OH)_3_)_0.1_·(HO_2_CC_6_H_4_CO_2_H)_0.7_.

Similarly, when amounts of CEST disodium salt and terephthalic acid were changed as 1.067 g (1.36 mmol), 0.4851 g (2.92 mmol), 2.134 g (2.72 mmol), and 0.3721 g (2.24 mmol) under the other identical conditions, respectively, the obtained CEST-linked Al-MIL-53 was denoted as **2** and **3**. Elemental analysis (wt%): for **2**: C, 46.78, H, 3.54, N, 0.13, Al, 8.5; for **3**: C, 27.57, H, 3.06, N, 0.56. Based on the molar ratio of the starting chemicals used and the results of the elemental analysis, the molecular structure of **2** is suggested as (HO)Al(O_2_CC_6_H_4_CO_2_)_0.9_(O_2_CCH_2_CH_2_Si(OH)_3_)_0.2_·(HO_2_CC_6_H_4_CO_2_H)_0.7_.

As a comparison, pure Al-MIL-53 (**4**) was also prepared according to the literature [20]. Elemental analysis (wt%): for as-made Al-MIL-53, C, 51.74, H, 2.45, N, 0.0, Al, 8.41; for activated Al-MIL-53 at 300 °C for 6 h, C, 45.9, H, 2.45, N, 0.0, Al, 12.9.

### 3.2. Preparation of Heterogeneous Catalysts Pd/CEST-Al-MIL-53 (***5***) and Pd/Al-MIL-53 (***6***)

Pd/CEST-Al-MIL-53 (**5**). To prepare 4 wt% of the Pd-loaded heterogeneous catalyst in the final product, the detailed procedure is as follows: In a 20 mL vial, 0.1892 g of CEST-Al-MIL-53 (**2**) was stirred in an aqueous solution of K_2_PdCl_4_ (7.4 mL, 10 mmol L^−1^) at ambient temperature for 20 min. A total of 2 mL of aqueous solution containing 0.0279 g of NaBH_4_ as a reductant was then added drop by drop under vigorous stirring. The resulting suspension was stirred at room temperature for 2 h, and black solid was separated using centrifugation at 2 × 10^4^ rotations per min (RPM) for 15 min. Then, water was used to wash the black product twice, and ethanol was used to wash the black product two times. Finally, the solid was put into a vacuum oven to dry at 65 °C overnight to afford the Pd-loaded heterogeneous catalyst Pd/CEST-Al-MIL-53 (**5**). Elemental analysis (wt%): C, 45.95, H, 2.81, N, 0.0. Metal contents from ICP analysis (wt%): Al: 7.89, Pd: 5.3.

Pd/Al-MIL-53 (**6**). When the same amount of Al-MIL-53 (**4**) activated at 300 °C was used to replace CEST-Al-MIL-53 during the preparation of **5** under the identical other conditions, the heterogeneous catalyst 6 was obtained and remarked as Pd/Al-MIL-53 (**6**). Elemental analysis (wt%): C, 39.17, H, 2.81, N, 0.11. Metal contents from ICP analysis (wt%): Al: 8.51, Pd: 7.0.

### 3.3. Characterization

A Bruker Advance D8 instrument using monochromatic CuKα radiation (λ = 1.5406 Å) was used to collect the low/wide-angle powder X-ray diffraction (PXRD) patterns in the 2θ range of 5–100° with a scan speed of 5. A Hitachi SU8030 operated at 30 kV was applied to record scanning/transmission electron microscope (S/TEM) images, and a JEOL JEM2100 instrument equipped with energy-dispersive X-ray (EDX) microanalytic apparatus was used and operated at a voltage of 160 kV to obtain high-resolution (HR)TEM images. All Diffuse Reflectance Infrared Fourier-Transform (DRIFT) spectra were measured on a Thermo Scientific Nicolet 6700 FTIR spectrometer with 256 scans and a resolution of 4 cm^−1^ in the range of 4000–400 cm^−1^, and KBr was used as a reference spectrum. X-ray photoelectron spectroscopies (XPS) were recorded on Thermo Scientific ESCALAB 250Xi. The sample was filled in a ZrO_2_ rotor with an inside diameter of 7 mm to perform the measurements of solid-state ^13^C and ^29^Si CP MAS NMR spectra at ambient temperature on a Bruker ASX 300 instrument equipped with magic angle spinning (MAS) hardware. A Netzsch STA 449F3 instrument (Selb, Germany) accompanied by a quartz crucible was used to perform the thermogravimetric analysis (TGA) in the temperature range of 20~1050 °C at a heating rate of 2 K per minute under Ar/O_2_/Ar atmosphere. The nitrogen adsorption–desorption isotherms were measured on an ASAP 2020 volumetric adsorption instrument from Micromeritics at 77.4 K [am(N_2_, 77 K) = 0.162 nm^2^] to calculate the specific Brunauer–Emmett–Teller (BET) surface area, Langmuir surface area, total pore volume, and microporous and mesoporous volume. Before nitrogen physisorption analysis, the sample needs to be degassed at expected temperatures, such as 120 °C, 250 °C, or 300 °C for 6 h under a vacuum pressure at least less than 10 μmHg. An Elementar Vario MICRO cube was used to measure the contents of elements C, N, and H. An inductively coupled plasma-optical emission spectrometer (ICP-OES) method was used to measure metals Pd and Al contents on the Thermo Scientific ICAP 7000 Series ICP spectrometer. A Perkin Elmer Clarus 580 gas chromatograph instrument equipped with a 30 m Velocity Wax capillary column was used to evaluate the catalytic performance of the catalyst using a gas chromatographic analysis.

### 3.4. Catalytic Activity of Heterogeneous Catalysts Pd/CEST-Al-MIL-53 (***5***) and Pd/Al-MIL-53 (***6***) in Suzuki–Miyaura Cross-Coupling Reaction

#### 3.4.1. The Optimization of Catalytic Reaction Conditions (Time, Dosage, Temperature, Solvent)

In a 100 mL three-neck round-bottom flask, phenylboronic acid (121.9 mg, 1 mmol) was dissolved in 10 mL of ethanol under stirring. Potassium carbonate (276.42 mg, 2 mmol) was then added. Then, 0.16 mL of decane (0.8 mmol) as the internal standard for the gas chromatography (GC) analysis was added. Afterwards, iodobenzene (102.0 mg, 0.5 mmol) or benzene derivatives containing halogen (0.5 mmol) and 5 mg catalyst **5** (2.49 × 10^−3^ mmol Pd, calculated by Pd content from ICP analysis) were then added to the mixture. The resulting suspension was stirred at 40 °C for the expected time. In this reaction system, to evaluate the catalytic activity, 5 mL of reaction suspension was taken at the expected time to centrifuge at the rotations of 20,000 per min for 10 min and obtain a supernatant for GC analysis before and after the addition of the catalyst. The results were used to optimize the reaction time.

When the reaction time was optimized as 10 h, the dosage experiment of catalyst **5** was explored. In this case, 2.5 mg and 10 mg of the catalyst were, respectively, used under the identical condition. The result showed that 5 mg of catalyst 5 is the best. Afterwards, the experiments to investigate temperature effect were performed under the identical other conditions. In terms of energy savings, 40 °C was chosen as the reaction temperature. Finally, based on the optimized reaction time, the temperature, catalyst dosage, and solvent effect were explored under the other identical conditions.

#### 3.4.2. Catalytic Activity of Heterogeneous Catalysts Pd/Al-MIL-53 (**6**) in Suzuki–Miyaura Cross-Coupling Reaction Under the Optimal Reaction Condition

As a comparison, 5 mg of catalyst **6** (3.29 × 10−^3^ mmol Pd) was used to perform the Suzuki–Miyaura C-C cross-coupling reaction under the optimal condition.

#### 3.4.3. Investigation on the Reusability of Catalysts **5** and **6**

Under the optional condition (5 mg of catalyst **5** or **6**, 40 °C, 10 h, ethanol), the reusability of catalyst **5** and **6** was investigated. In this step, after every catalytic reaction, the solid was collected by centrifugation and washed with ethanol four times, then with water twice, with ethanol twice again, and dried in a vacuum oven at 65 °C overnight. The obtained catalyst was used for repeated catalytic reactions.

#### 3.4.4. Pd/CEST-Al-MIL-53 Catalyzed the Reaction of Phenylboronic Acid with Halogen-Substituted Benzene Derivatives Under the Identical Optimal Condition

In this reaction, only the equivalent halogen-substituted benzene derivative was used to replace iodobenzene in the above-mentioned reaction. All procedures are completely same under the identical condition.

For all the above-mentioned experiments, after the catalytic reaction, the suspension was centrifuged at the rotations of 2 × 10^4^ per min for 10 min, and the supernatant was filtrated to obtain a clear ethanol solution. The solid was washed with ethanol four times, and the centrifuged ethanol solution was filtrated. All ethanol solutions were combined together, and the ethanol solvent was removed under the vacuum condition to afford a solid. Then, crude catalytic product was extracted by diethyl ether from the solid at least three times. All the extracted diethyl ether solutions were combined together, and the solvent diethyl ether was removed to obtain the solid again. Finally, hexane was used to extract the product from the solid at least four times. After hexane was removed from the combined hexane-extracted liquid, the isolated solid product was obtained, which was characterized using ^1^H NMR spectroscopy, to determine the isolated product yield.

#### 3.4.5. Hot Filtration Experiment

For the Pd/CEST-Al-MIL-53-catalyzed reaction of phenylboronic acid (121.9 mg, 1 mmol) with iodobenzene (102.0 mg, 0.5 mmol) in 10 mL of ethanol in the presence of potassium carbonate (276.42 mg, 2 mmol) and 0.16 mL of decane, after a reaction at 40 °C for 1 h, the catalyst was completely removed by centrifugation and filtration to obtain a clear solution. Such a solution was stirred at 40 °C for 12 h. Then, 5 mL of the solution was taken for the GC analysis and Pd leaching test.

Similarly, the same reaction was run for 1 h to obtain a clear solution. The equivalent amounts of substrates and base were then added into this clear solution and stirred at 40 °C for 12 h. A total of 5 mL of the mixture was taken, centrifugated, and filtered to obtain a clear solution for the GC analysis.

## 4. Conclusions

Three CEST-functionalized Al-MIL-53 samples were successfully fabricated using the hydrothermal reaction of aluminum nitrate, terephthalic acid, and CEST disodium salt by adjusting the molar ratio of CEST to terephthalic acid under an identical molar ratio of Al^3+^/-COOH of 1:1. Their composition, morphology, and structure were characterized via the IR spectroscopy, NMR spectroscopy, electron microscopy, XRD, and N_2_ physisorption. With the increasing CEST content, CEST-Al-MIL-53 still preserves an Al-MIL-53-like structure. Such a functionalized MOF was used as the support to prepare the CEST-Al-MIL-53-supported Pd catalyst for the Suzuki–Miyaura C-C cross-coupling reaction of aryl halides and phenylboronic acid in the presence of base. Note that the microporous catalyst Pd/CEST-Al-MIL-53 indicated a good catalytic activity and a high reusability in comparison to Pd/Al-MIL-53, which is attributed to the nanofibrous structure of the silicon-species-integrated CEST-Al-MIL-53. During the catalytic reaction, this kind of nanofiber microstructure can be converted into 3D cross-networks and enables the leachable Pd particles after completion of transformation to orientally redeposit and inlay into these networks as the monodisperse spheres, thereby preventing them from aggregation and leaching. As a result, the catalyst Pd/CEST-Al-MIL-53 (**5**) showed a high catalytic performance, long-term stability, and reusability. In addition, the catalytic performance is strongly influenced by the catalyst dosage, temperature, time, solvent, and the type of the substituted group on the benzene ring. This work further extends the catalytic application of hybrid metal–organic frameworks.

## Figures and Tables

**Table 1:** Specific surface area and pore parameters of CEST-Al-MIL-53 (**1**–**3**), Al-MIL-53 (**4**), Pd/CEST-Al-MIl-53 (**5**), and Pd/Al-MIL-53 (**6**).

Sample	T ^a^ (°C)	*S*_L._ ^b^ (m^2^ g^−1^)	*S*_BET_ ^c^ (m^2^ g^−1^)	V_s. p._ ^d^ (cm^3^ g^−1^)	*V*_micro._ ^e^ (cm^3^ g^−1^)	*V*_meso._ ^f^ (cm^3^ g^−1^)
CEST-Al-MIL-53 (**1**)	250 ^g^	78	55	0.058	0.015	0.023
CEST-Al-MIL-53 (**2**)	250 ^g^	303	216	0.192	0.080	0.078
CEST-Al-MIL-53 (**2**)	250 ^h^	427	306	0.239	0.122	0.078
CEST-Al-MIL-53 (**3**)	250 ^g^	195	138	0.129	0.041	0.048
Al-MIL-53 (**4**)	250 ^h^	715	515	0.274	0.240	0.014
Al-MIL-53 (**4**)	300 ^h^	1629	1154	0.571	0.577	0.011
Pd/CEST-Al-MIL-53 (**5**)	120 ^h^	340	241	0.189	0.077	0.057
Pd/Al-MIL-53 (**6**)	250 ^h^	869	624	0.512	0.255	0.145

Note: ^a^ Activated temperature under high vacuum. ^b^ Langmuir surface area. ^c^ Specific BET surface area. ^d^ Single-point pore volume at relative pressure *P*/*P*_0_ = 0.99. ^e^ Micropore volume (pore diameter is less than 2 nm). ^f^ Mesopore volume (pore diameter ranges from 2 nm to 50 nm. ^g^ Sample was degassed at expected temperature for 2 h. ^h^ Sample was degassed at expected temperature for 12 h.

**Table 2:** Catalytic performance of Pd/CEST-Al-MIL-53 (**4**) for Suzuki–Miyaura cross-coupling reaction of iodobenzene and phenylboronic acid based on catalyst dosage, reaction time, reaction temperature, and solvent.

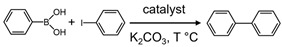							
Dosage (mg)	Ratio ^a^	Ratio ^b^	Time ^c^ (h)	T ^d^ (°C)	Solvent	Conversion ^e^ (%)	Yield (Isolated, %)
2.5	2.0	2.0	10	40	ethanol	74	n.d. ^f^
5.0	2.0	2.0	10	40	ethanol	97	96
10.0	2.0	2.0	10	40	ethanol	97	96
5.0	2.0	2.0	0.5	40	ethanol	14.5	n.d. ^f^
5.0	2.0	2.0	1	40	ethanol	29	n.d. ^f^
5.0	2.0	2.0	1.5	40	ethanol	43	n.d. ^f^
5.0	2.0	2.0	2	40	ethanol	57	n.d. ^f^
5.0	2.0	2.0	3	40	ethanol	67	n.d. ^f^
5.0	2.0	2.0	4	40	ethanol	76	n.d. ^f^
5.0	2.0	2.0	5	40	ethanol	86	n.d. ^f^
5.0	2.0	2.0	9	40	ethanol	94	n.d. ^f^
5.0	2.0	2.0	10	25	ethanol	63	n.d. ^f^
5.0	2.0	2.0	5	60	ethanol	>99	97
5.0	2.0	2.0	2	80	ethanol	>99	97
5.0	2.0	2.0	10	40	water	31	n.d. ^f^
5.0	2.0	2.0	10	40	methanol	91	87
5.0	2.0	2.0	24	40	hexane	2	n.d. ^f^
5.0	2.0	2.0	10	40	THF	0	n.d. ^f^

Note: ^a^ Molar ratio of phenylboronic acid to iodobenzene. ^b^ Molar ratio of potassium carbonate to phenylboronic acid. ^c^ Reaction time. ^d^ Reaction temperature. ^e^ Result from GC analysis. ^f^ Not determined.

**Table 3:** A comparison of the catalytic performance of Pd-loaded metal–organic frameworks (Pd-MOFs) catalysts for the Suzuki–Miyaura cross-coupling reaction of iodobenzene and phenylboronic acid in the presence of K_2_CO_3_.

Catalyst	Dosage ^a^ (mmol)	*T* (°C), *t* (h)	Conversion/Yield	Recycling Run No.	Reference
Pd/CEST-Al-MIL-53	5.0	40, 10	97/96 ^b,^ 97/96 ^c^	5	This work
Pd/Al-MIL-53	6.6	40, 10	93/93 ^b^, 31/31 ^c^	4	This work
Pd/MIL-53(Al)-NH_2_	5.0	40, 0.5	99/94	-	[37]
Pd@UIO-66	0.94	30, 0.5	-/95 ^b^, -/80 ^c^	5	[44]
Pd(II)@MOF-253	20	120, 10	99/14 ^b^	-	[45]
Pd/UIO-66-NH_2_	2.5	60, 1.33	-/68 ^b^	-	[46]
Pd-Sr-MOF-1-PI	0.35	80, 2	-/100 ^b^, -/95 ^c^	5	[47]
Pd-Cu-MOF	4.6	60, 12	-/99 ^b^, -/98 ^c^	4	[48]

Note: ^a^ Dosage is Pd content in the catalyst, which corresponds to 1 mol of substrate iodobenzene. ^b^ Conversion and yield for first run. ^c^ Conversion and yield for last recycling run.

**Table 4:** Suzuki–Miyaura C-C cross-coupling reaction of phenylboronic acid with aryl halides with substitute group over Pd/CEST-Al-MIL-53 (**5**).

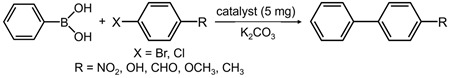				
Entry	R	R_1_	Conversion ^a^	Yield (%)
1	Br	NO_2_	100	98 ^b^
2	Br	OH	100	95 ^c^
3	Br	CHO	55.7	n.d.
4 ^d^	Br	CHO	100	98 ^c^
5	Br	OCH_3_	12.3	n.d.
6 ^d^	Br	OCH_3_	34	n.d.
7	Br	CH_3_	15.1	n.d.
8	Cl	NO_2_	4.5	n.d.

Note: ^a^ GC analysis. ^b^ Isolated yield. ^c^ NMR yield. ^d^ Reaction temperature (80 °C) and time (6 h).

## Data Availability

No further data are available.

## Supplementary Materials

The following supporting information can be downloaded at: https://www.mdpi.com/article/10.3390/molecules30030656/s1, Figure S1: (a) SEM and (b) TEM images of sample Pd/Al-MIL-53 (**6**); Figure S2: ^1^H NMR of product biphenyl; Figure S3: ^1^H NMR of product 4-nitrobiphenyl; Figure S4: ^1^H NMR of product 4-hydroxybiphenyl; Figure S5: ^1^H NMR of product 4-phenylbenzaldehyde; Figure S6: ^1^H NMR of product 4-methoxybiphenyl; Figure S7: ^1^H NMR of product 4-methylbiphenyl.
