# Supplementary material for: Carboxyethylsilanetriol-Functionalized Al-MIL-53-Supported Palladium Catalyst for Enhancing Suzuki–Miyaura Cross-Coupling Reaction
Source: Molecules. 2025 Feb 1;30(3):656. doi: 10.3390/molecules30030656 (PMC11820489; doi:10.3390/molecules30030656)
Supplement: Supplementary file 1 [file molecules-30-00656-s001.zip › molecules-3420934-supplementary.pdf]

Supporting Information for

Carboxyethylsilanetriol-Functionalized Al-MIL-53-Supported Palladium Catalyst for Enhancing Suzuki-Miyaura Cross-Coupling Reaction

Yucang Liang *, Xin Ning †, and Yanzhong Zhen ‡

Institut für Anorganische Chemie, Eberhard Karls Universität Tübingen, Auf der Morgenstelle 18, 72076 Tübingen, Germany

† Present address: Jiangsu Key Laboratory of Environmental Material and Engineering, School of Environmental Science and Engineering, Yangzhou University, Yangzhou 225000, P. R. China

‡ Present address: Research Institute of Comprehensive Energy Industry Technology, College of Chemistry & Chemical Engineering, Yan’an University, No. 580, Shengdi Road, Baota District, Yan'an 716000, Shaanxi, China

* Corresponding author

Dr. Yucang Liang, Email: yucang.liang@uni-tuebingen.de

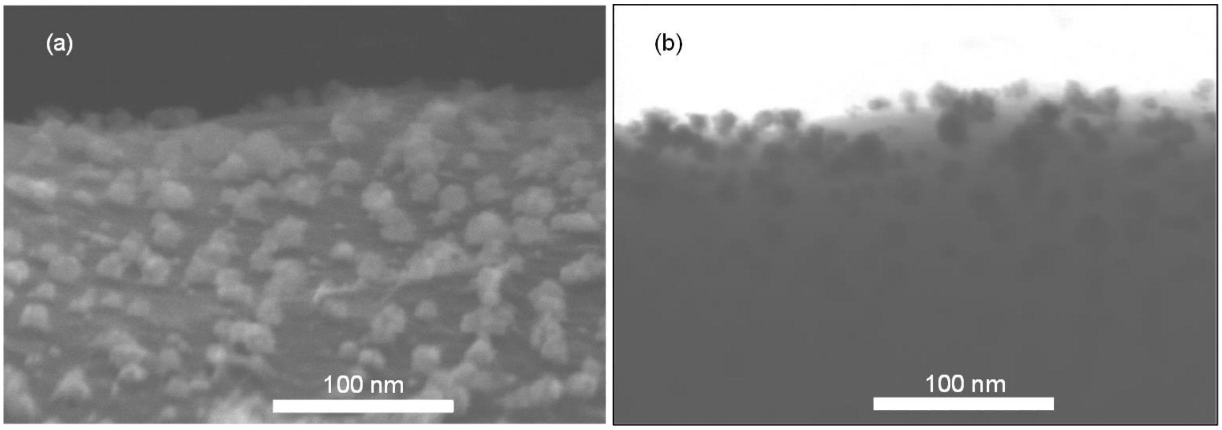

Figure S1. (a) SEM and (b) TEM images of sample Pd/Al-MIL-53 (**6**).

^{1}H NMR data for products

biphenyl (Table 2): ^{1}H-NMR (400 MHz, $CDCl_3$, 25 ℃): δ 7.61-7.58 (d, 4H), 7.47-7.42 (t, 4H), 7.37-7.33 (t, 2H).

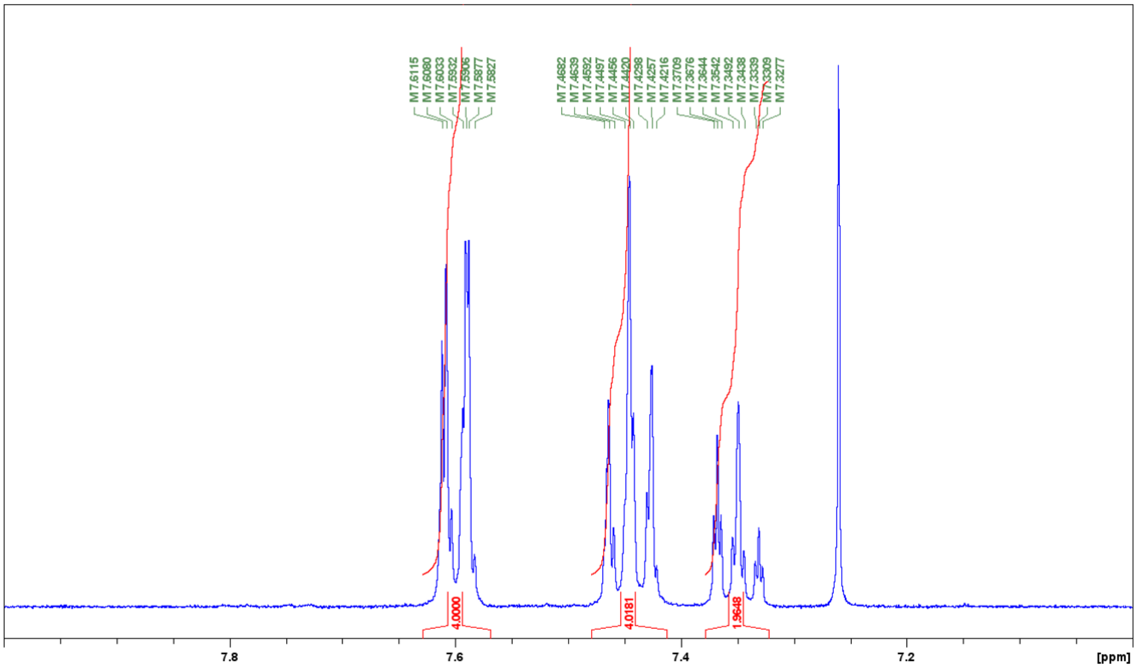

Figure S2. ^{1}H NMR of product biphenyl

4-nitrobiphenyl: ^{1}H NMR (400 MHz, $CDCl_3$, 25 °C): δ 8.32-8.29 (d, 2H), 7.75-7.73 (d, 2H), 7.64-7.62 (d, 2H), 7.52-7.45 (m, 3H).

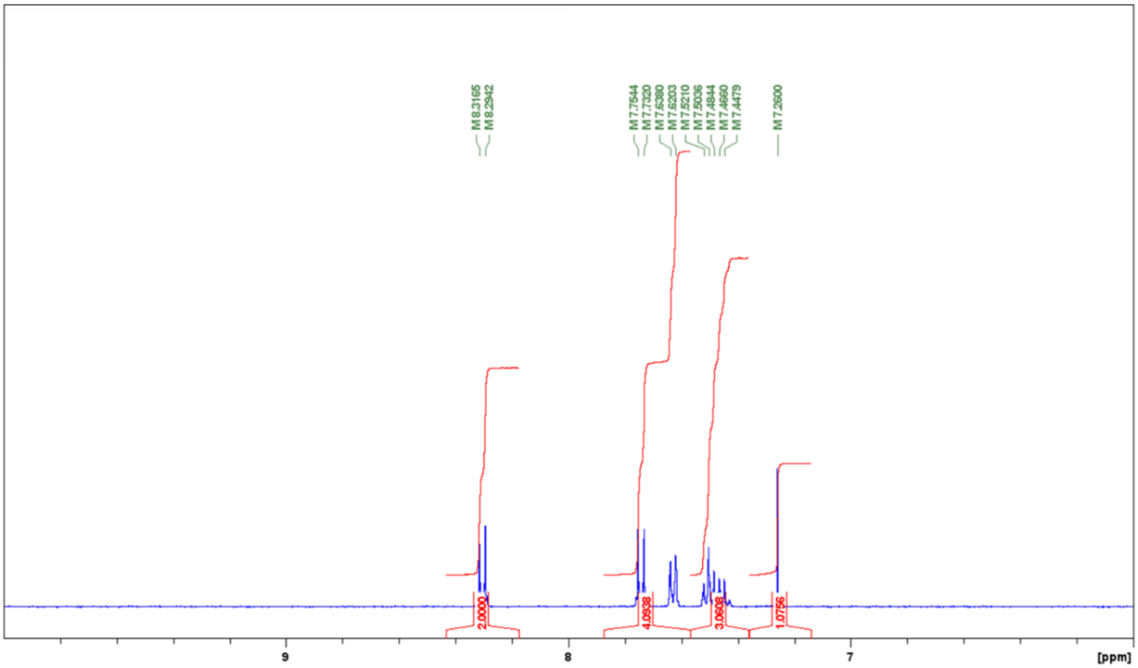

Figure S3. ^{1}H NMR of product 4-nitrobiphenyl

4-hydroxybiphenyl (biphenyl-4-ol): ^{1}H NMR (400 MHz, $CDCl_3$, 25 °C): δ 7.63-7.59 (d, 2H), 7.55-7.50 (d, 2H), 7.43-7.40 (t, 2H), 7.32-7.29 (t, 1H), 6.92-6.90 (d, 2H), 4.79 (s, 1H).

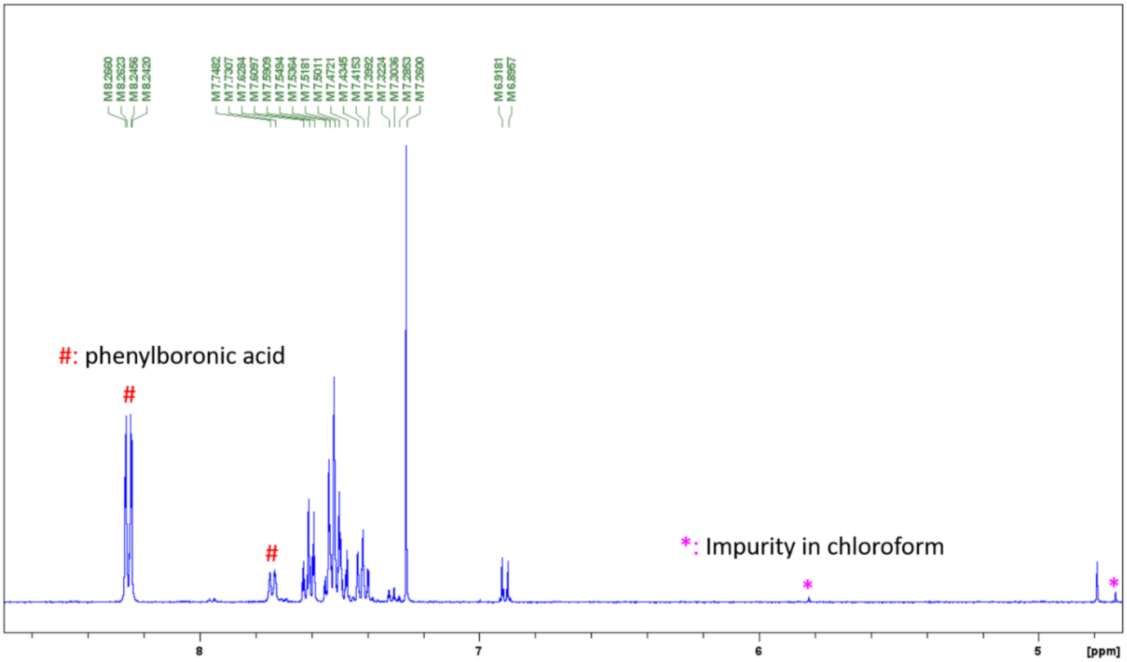

Figure S4. ^{1}H NMR of product 4-hydroxybiphenyl

4-phenylbenzaldehyde: ^{1}H NMR (400 MHz, $CDCl_3$, 25 °C): δ 10.06 (s, 1H), 7.97-7.95 (d, 2H), 7.77 - 7.75 (d, 2H), 7.66-7.63 (d, 2H), 7.51-7.40 (m, 3H).

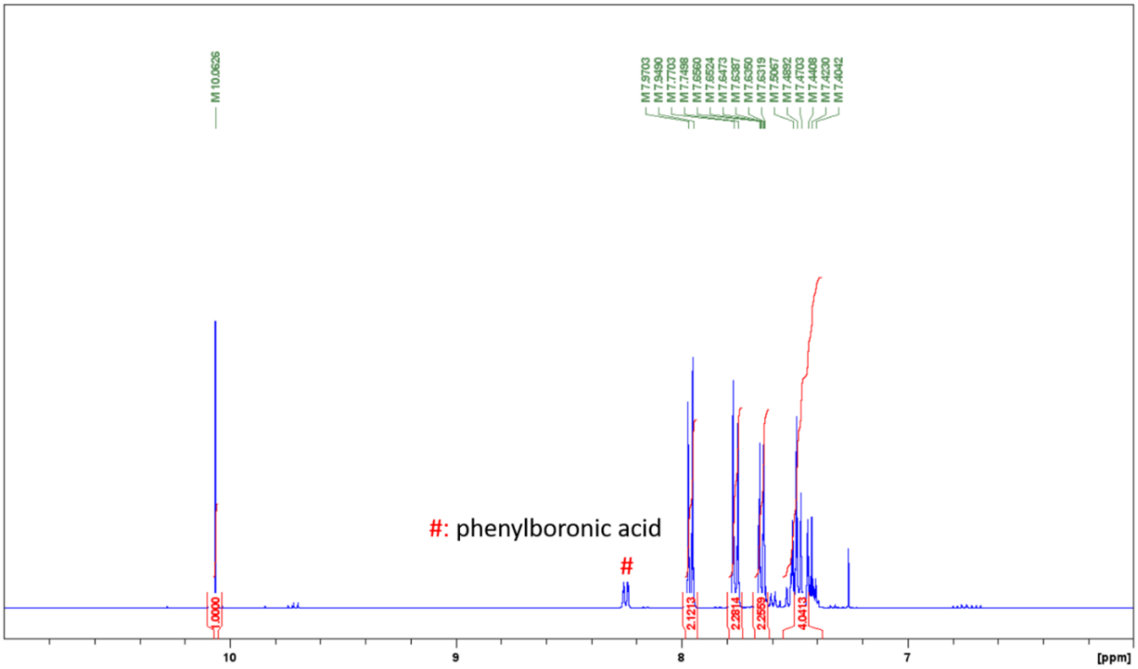

Figure S5. ^{1}H NMR of product 4-phenylbenzaldehyde

4-methoxybiphenyl: ^{1}H NMR (400 MHz, $CDCl_3$, 25 °C): δ = 7.56-7.52 (m, 4H), 7.43-7.40 (m, 2H), 7.32-7.28 (m, 1H), 6.99-6.97 (d, 2H), 3.86 (s, 3H).

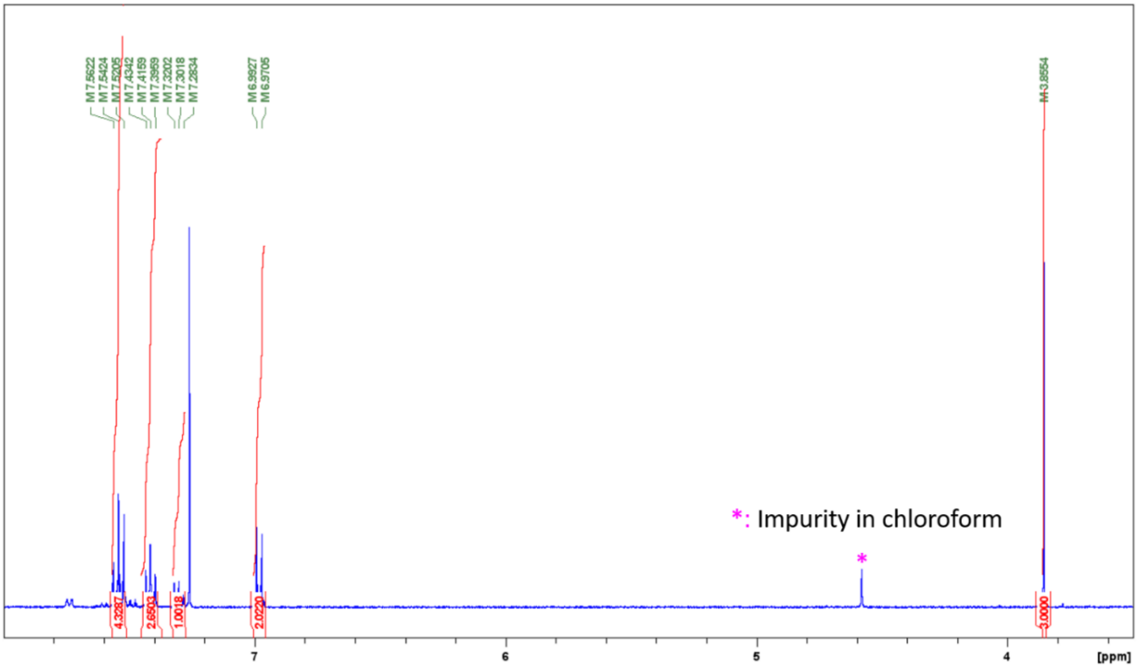

Figure S6. ^{1}H NMR of product 4-methoxybiphenyl

4-methylbiphenyl: ^{1}H NMR (400 MHz, $CDCl_3$, 25 ℃): δ = 7.59-7.57 (d, 2H), 7.51-7.49 (d, 2H), 7.44-7.41 (t, 2H), 7.34-7.32 (t, 1H), 7.24 (d, 2H), 2.40 (s, 3H).

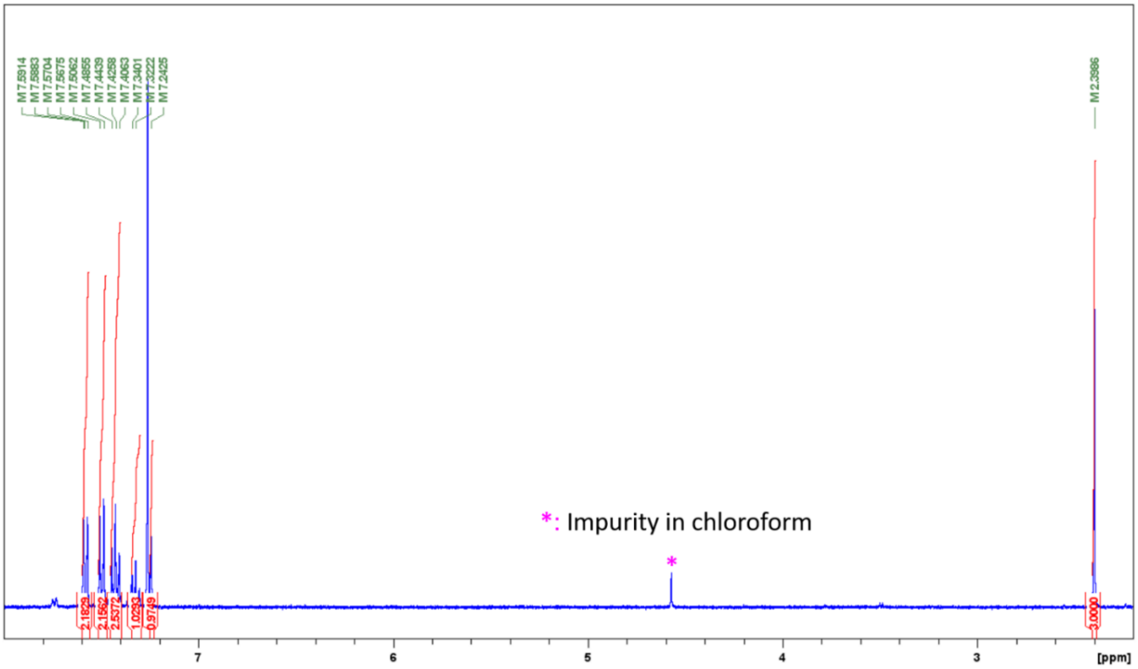

Figure S7. ^{1}H NMR of product 4-methylbiphenyl
